# Tick tock: how the body’s circadian clock keeps cardiometabolic rhythm in check

**DOI:** 10.1042/CS20250046

**Published:** 2026-11-04

**Authors:** Masaaki Sato, Abdulaziz M.A. Alammar, Frédéric Gachon, Daniel Mauvoisin, Denuja Karunakaran

**Affiliations:** 1 Cardiometabolic Immunity Laboratory, Department of Physiology, Cardiovascular Disease and Metabolism, Diabetes & Obesity Programs, Biomedicine Discovery Institute (BDI) and Victorian Heart Institute (VHI), Monash University, Level 2, Victorian Heart Hospital, 631 Blackburn Road, Clayton, Victoria 3168, Australia; 2 Department of Biomedicine, Aarhus University, Høegh-Guldbergs Gade 10, 8000 Aarhus C, Denmark; 3 Steno Diabetes Center Aarhus, Palle Juul-Jensens Boulevard 11, 8200 Aarhus N, Denmark; 4 Nantes Université, CNRS, Inserm, l'institut du thorax, F-44000 Nantes, France

**Keywords:** cardiometabolic diseases, chronobiology, chronodisruption, chronotherapeutics, circadian rhythms, clock genes

## Abstract

The circadian clock orchestrates the internal timing of numerous physiological processes through an approximately 24-h cycle. It comprises a hypothalamic central clock, which responds to light cues and entrains peripheral clocks within individual cells and organs that, depending on the tissue, regulate gene expression across diverse biological functions in a tissue-specific manner. Peripheral clocks are also entrained by feeding and exercise-related cues, enabling their synchronization with feeding rhythm and physiology. Together, these clocks maintain a complex molecular network essential for metabolic homeostasis. Disruption of circadian rhythms, driven by genetic, environmental, or behavioral factors, is increasingly recognized as a major contributor to cardiometabolic disorders. Modern lifestyles, including shift work, irregular sleep, prolonged exposure to artificial light at night, jet lag, and erratic eating schedules, misalign circadian clocks with environmental cues, contributing to disruption of circadian rhythms or chronodisruption. This misalignment alters hormonal regulation, promotes systemic inflammation, and impairs metabolic balance, thereby increasing the risk of cardiovascular disease, obesity, type 2 diabetes, and related complications. The present review examines the mechanistic roles of core circadian clock genes in cardiometabolic health, highlighting their regulation of physiological processes in peripheral organs such as the heart, liver, and adipose tissue. We further discuss how circadian clock disruption accelerates disease onset and progression and explore emerging therapeutic strategies, including pharmacological modulation of the molecular circadian clock components and personalized chronotherapy. These approaches offer promising avenues to restore circadian alignment and mitigate cardiometabolic risk.

## Introduction

The circadian clock, named after the Latin words ‘circa’ and ‘diem’ meaning about a day, is a molecular endogenous time-keeping system that entrains internal rhythms in organisms from bacteria to plants and mammals to the light–dark cycles caused by Earth’s rotation [1]. In mammals, the central clock is composed of two bilateral groups of neurons located in the suprachiasmatic nuclei (SCN) of the hypothalamus. As it primarily responds to the dominant zeitgeber time (ZT, i.e. time-giver) light [1,2], this central clock orchestrates daily rhythms in physiology, behavior and metabolism. However, peripheral clocks present in virtually every cell also respond to non-photic ZTs such as nutrients, hormones, temperature fluctuations, exercise, and feeding cues.

Molecular timing is set by core clock proteins—CLOCK (circadian locomotor output cycles kaput), BMAL1 (brain and muscle aryl hydrocarbon receptor nuclear translocator-like protein 1, official gene name *ARNTL*), NPAS2 (neuronal Per-Arnt-Sim (PAS) domain protein 2), PER (PERIOD1–3), CRY (CRYPTOCHROME1–2), REV-ERB (reverse-erythroblastosis virus α/β), and ROR (retinoic acid receptor-related orphan receptor)—that form transcriptional–translational feedback loops (TTFLs) to drive rhythmic gene expression [3]. The TTFL features a positive limb, in which CLOCK–BMAL1 or NPAS2–BMAL1 heterodimers bind E-box elements to activate *PER1/2/3* and *CRY1/2* [3]; and a negative limb, in which PER–CRY complexes accumulate, undergo phosphorylation by casein kinase Iδ/ε (CKIδ/ε), translocate to the nucleus, and repress BMAL1 heterodimer activity (Figure 1) [4]. Rhythmic ubiquitin-proteasome-mediated degradation of PER–CRY resets the system and initiates the next 24-h cycle [5,6].

**Figure 1:**
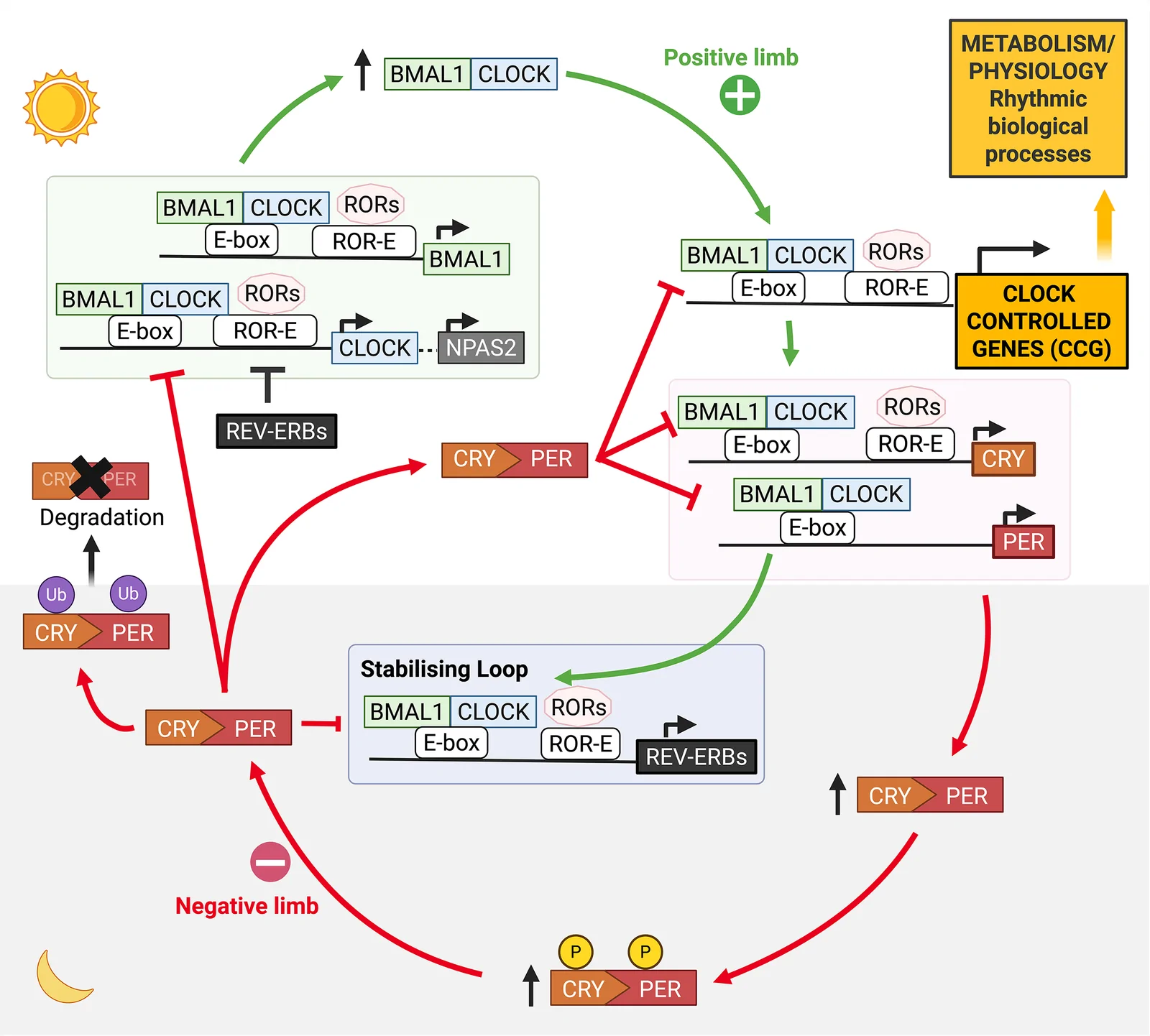
General TTFLs of the mammalian circadian clock In the core-positive limb (green, ‘+’), BMAL1/CLOCK or BMAL1/NPAS2 heterodimers bind E-box elements to drive transcription of clock-controlled genes (CCGs), including *PER* and *CRY*. CRY-PER dimers accumulate and translocate to the nucleus, where they repress BMAL1-CLOCK/NPAS2 activity at E-box-containing promoters, forming the primary negative feedback loop (red, ‘−’). The CRY-PER complex is progressively phosphorylated (yellow ‘P’), ubiquitinated (purple ‘Ub’), and degraded via the proteasome, relieving repression and permitting renewed BMAL1-dependent transcription. A secondary stabilizing loop involves REV-ERBs, which repress *BMAL1* and *CLOCK* transcription, and RORs, which activate it, fine-tuning oscillation amplitude and phase. Together, these interlocking loops generate robust ∼24-h rhythms in CCG expression and downstream rhythmic metabolism and physiology.

A secondary ‘stabilizing’ loop is formed by the nuclear receptors REV-ERBα/β (also known as NR1D1 and NR1D2) and RORα/γ. REV-ERBα/β represses several core clock genes, notably *BMAL1*, *CLOCK*, and *CRY1*, via co-repressors such as NCoR and HDAC3 [7–9], while RORα/γ promotes their expression via the co-activator SRC-2 [10,11], together reinforcing circadian amplitude and stabilizing the circadian phase. This dynamic push–pull system maintains high-fidelity *Bmal1* rhythms, limiting phase drift and preserving rhythmicity despite environmental and metabolic fluctuations. Accordingly, this loop is essential for clock function: combined deletion of *Rev-erbα* and *Rev-erbβ* causes circadian arrhythmicity in mice [7,12]. Beyond the clock, REV-ERBs also coordinate broader circadian physiology and metabolism [13].

Circadian disruption—via shift work, mistimed eating/sleeping, stress, or environmental misalignment—causes chronodisruption, associated with metabolic syndrome, cardiovascular disease, immune dysfunction, neurodegeneration, and cancer. Acute circadian misalignment, as in jet lag, elevates postprandial glucose, insulin, and mean arterial pressure and perturbs cortisol and leptin rhythms in humans [14]. Interestingly, rotating night-shift schedules that disrupt circadian rhythms more frequently and unpredictably create greater metabolic dysfunction (e.g. increased diabetes risk), rather than fixed night work [15].

Focusing on cardiometabolic phenotypes, the present review examines how core clock components coordinate glucose and lipid metabolism, blood pressure, and vascular function across key metabolic tissues (e.g. liver, heart, and adipose tissue). We briefly summarize the molecular architecture and entrainment mechanisms of the circadian clock to provide mechanistic context, then discuss how circadian disruption contributes to insulin resistance, dyslipidemia, hypertension, and cardiovascular disease. We also highlight emerging chronotherapeutic and lifestyle strategies aimed at restoring circadian alignment and improving metabolic health. Key circadian terms are defined in Table 1. Owing to space constraints, we regret that not all relevant studies could be discussed. It is noteworthy that most mechanistic clock-gene knockout studies discussed below were conducted exclusively in male animals, with sex often unreported, unless otherwise indicated. This male-biased evidence base is a substantial gap in the field [16], addressed further in the ‘Sex differences in chronobiology’ section.

**Table 1:** Summary of circadian clock terminology

Term	Definition
**Circadian clock**	Endogenous molecular biological timekeeping system that generates ∼24-h cycles in physiology and behavior
**Circadian rhythm**	Endogenous biological rhythm within a ∼24-h period that occurs in constant conditions
**Diurnal rhythms**	Biological rhythms that follow a ∼24-h cycle driven or synchronized by external cues such as light
**Amplitude**	A measure of the level of gene expression from the midline to either the peak or the trough
**Period**	Length of time it takes to complete one full cycle of the circadian rhythm, typically ∼24 h
**Phase**	Timing of a specific moment (e.g. peak, trough, or a midpoint) in a circadian rhythm
**Acrophase (Peak)**	Timing of the highest point in the circadian cycle
**Nadir (Trough)**	Timing of the lowest point in a circadian cycle
**Mesor**	Midline Estimating Statistic Of Rhythm: estimated using a cosine function, the mesor represents the mean value based on the distribution of values across the cycles of the circadian rhythm
**Central clock or pacemaker (SCN)**	The SCN are two small bilateral regions of the hypothalamus that sit on top of the optic chiasm in the anteroventral region. SCN synchronize peripheral clocks and thus commonly referred to as the central clock or circadian pacemaker
**Pacemaker**	An oscillator that sets the pace for other oscillators or drives rhythmic outputs. For example, SCN keep time independently and synchronize peripheral clocks based on environmental cues
**Oscillator**	A set of components that work together to create a regular, repeating rhythm with a definable period length. For example, the CLOCK–BMAL1–PER–CRY loop in cells acts as an oscillator, creating a ∼24-h rhythmic cycle
**Entrainment**	The process by which an environmental cue, such as the light–dark cycle, regulates the period and phase of a self-sustained oscillator
**Peripheral clocks**	Tissue-specific oscillators in organs (e.g. liver, heart, adipose tissue)
**TTFL**	Transcriptional–translational feedback loop driving rhythmic gene expression
**Zeitgeber**	External/internal cues (‘time givers’) that entrain the clock. e.g. light, feeding, exercise
**Core clock genes**	Set of genes that form the molecular basis of the circadian clock over a 24-h timeframe. Main loop: Activators: *CLOCK*, *BMAL1*, *NPAS2*; Repressors: *PER*, *CRY* Stabilizing loop: *REV-ERBα/β* (Nuclear Receptor Subfamily 1 Group D Member 1 and 2) and *RORα/β/γ* (RAR-Related Orphan Receptor Alpha and Beta and Gamma)
**Auxiliary regulators**	Function to fine-tune and optimize clock output. Examples include: E4BP4 (E4 binding protein 4; also known as NFIL3) influences circadian output gene expression and plays an important role in immune function PARbZIP (proline and acidic amino acid-rich basic leucine zipper proteins)—clock-regulated transcription factors that activate D-box elements in clock output genes CKIε/δ—dual role: (i) stabilizes or (ii) redirects to proteasomal degradation of PER and CRY proteins via phosphorylation at different sites SIRT1 (Sirtuin 1)—an NAD^+^-dependent deacetylase that modulates CLOCK–BMAL1 activity and links circadian timing to cellular energy status AMPK (AMP-activated protein kinase)—phosphorylates CRY, integrating energy sensing with circadian regulation GSK3β (glycogen synthase kinase 3 beta)—stabilizes PER and REV-ERB proteins, influencing phase and amplitude of rhythms NAD^+^/NADH (redox regulators)—regulate CLOCK–BMAL1 transcriptional activity via metabolic state-dependent feedback
**CCGs**	Genes regulated by the circadian clock encoding proteins involved in physiological functions, including metabolic and cardiovascular output
**Circadian disruption/misalignment**	Misalignment of central and/or peripheral internal clock rhythmicity (e.g. amplitude, phase, period, etc.)

## Molecular architecture of the circadian clock

This section provides a concise overview of circadian clock organization as a framework for understanding cardiometabolic phenotypes discussed later in the review. The mammalian clock system is hierarchical: characterized via lesion and transplantation experiments, the SCN orchestrate 24-h drinking behavior and locomotor activity [17–19]. The SCN comprise a ventrolateral core and a dorsomedial shell with ∼20,000 neurons exhibiting membrane potential rhythm synchronizing peripheral oscillators via rhythmic firing and neuropeptide release [1,2]. SCN neurons express several signaling peptides, including vasoactive intestinal polypeptide (VIP) and arginine vasopressin (AVP). VIP is synthesized by SCN core neurons and induces PER1/2 to maintain network synchrony within the SCN [1,20]. AVP, produced predominantly by SCN shell neurons, helps regulate the SCN’s overall circadian period and contributes to inter-neuronal coupling that stabilizes network rhythmicity. Systemic timing is propagated via autonomic outputs that regulate cardiovascular tone, gastrointestinal motility, and thermoregulation. The regulation of rhythmic secretion of humoral signals such as glucocorticoids [21] via the hypothalamic–pituitary–adrenal (HPA) axis and insulin from pancreatic β-cells [22,23] contributes to peripheral clock alignment in metabolic tissues [24–26]. Loss of SCN control leads to the loss of synchronization of peripheral circadian clocks and leads to progressive desynchrony of peripheral TTFLs and CCGs [25,27,28] regulating glucose, lipid, and vascular homeostasis, ultimately contributing to metabolic dysfunction, hypertension, and increased cardiovascular risk.

### Photic entrainment

Light is the principal central clock ZT [2] (Figure 2). Intrinsically photosensitive retinal ganglion cells expressing the photopigment melanopsin (OPN4) detect ambient light and project via the retinohypothalamic tract [29,30]. OPN4 ablation impairs photoentrainment in mice [31] while loss of all three photopigments—rods, cones, and melanopsin—completely abolishes it [32]. At SCN synapses, glutamate and pituitary adenylate cyclase-activating polypeptide activate N-methyl-D-aspartate receptors, raising calcium and engaging CaMKII and MAPK, which in turn phosphorylate cAMP response element-binding protein to induce *Per* gene transcription and reset phase [33,34]. Morning light advances the circadian phase via PER1, while evening light delays the phase via *Per2* transcription and stability (reviewed in [34,35]).

**Figure 2:**
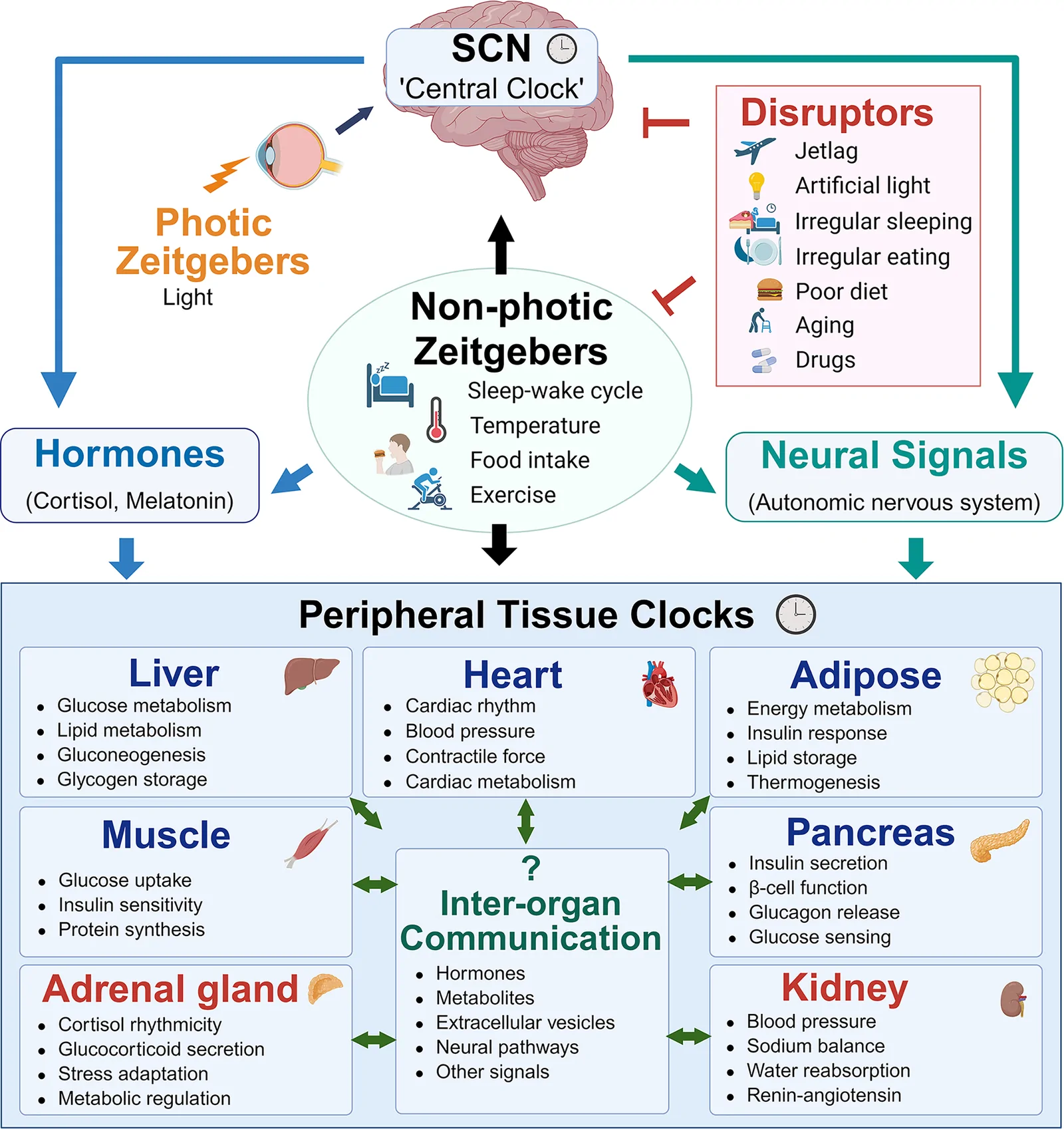
Central and peripheral entrainment by photic and non-photic ZTs The SCN function as the central circadian pacemaker and are primarily entrained by photic cues conveyed by the light–dark cycle. Non-photic ZTs, including sleep–wake behavior, temperature, food intake, and physical activity, modulate circadian timing predominantly via hormonal (cortisol, melatonin) and autonomic (neural) pathways. Circadian disruptors such as jet lag, artificial light exposure, irregular sleep or eating patterns, poor diet, aging, and pharmacological agents can impair these entrainment mechanisms. Hormonal and neural signals relay timing information from the SCN to peripheral clocks in metabolic and cardiovascular tissues (liver, skeletal muscle, heart, adipose tissue, pancreas, kidney, and adrenal gland), which coordinate tissue-specific rhythmic processes, including glucose and lipid metabolism, insulin secretion, cardiac function, thermogenesis, renal homeostasis, glucocorticoid/aldosterone secretion, and blood pressure rhythm. Peripheral clocks may also communicate with one another (inter-organ communication) via hormones, metabolites, and neural pathways, the mechanisms of which remain incompletely defined.

### Non-photic entrainment

Feeding, exercise, temperature, and sleep–wake cues can entrain the circadian clock (Figure 2), especially in peripheral tissues. In rodents, mistimed feeding during the resting phase shifts the circadian clock in liver and white adipose tissue (WAT) by up to 12 h and almost abolishes the rhythm in muscle and kidney [36–40], while not affecting the SCN or lung rhythms [37,39,41]. In the liver, shifts accrue gradually after food inversion, and SCN lesions accelerate feeding-driven entrainment [28,42], implying that an intact SCN counteracts the entrainment of the liver clock by mistimed feeding—a process involving glucocorticoids [43]. Other tissues, especially skeletal muscles, are entrained by the timing of physical activity and exercise [41,44]. Temperature cycles also contribute to the entrainment of peripheral oscillators via the heat-shock factor 1 [45,46]. Modern lifestyle factors such as shift work, social jet lag, exposure to artificial light at night, mistimed eating and exercise, sleep loss, rapid travel across time zones, and seasonal environmental changes can disrupt circadian entrainment pathways. Further, aging and certain medications, including corticosteroids and β-blockers, may further impair circadian alignment (Figure 2). Together, these disruptions are associated with an increased risk of cardiometabolic and mood disorders, underscoring the clinical importance of maintaining circadian synchrony (reviewed in [47]).

### Circadian temporal dynamics of the genome

Whole-genome transcriptomics and proteomics show that thousands of mRNA protein-coding genes and proteins, including post-translationally modified isoforms, display tissue-specific diurnal or circadian rhythms. These rhythms are defined by three parameters (Figure 3 and Table 1):

- Period: duration or length of 1 complete cycle, typically ∼24 h for circadian rhythms;
- Acrophase: timing of peak expression relative to a reference point (e.g. lights-on);
- Amplitude: size of expression difference between peak (acrophase) and trough (nadir).

Together, they form each gene’s rhythmic signature and coordinate cellular temporal timing and processes. Circadian regulation far extends beyond circadian core clock genes to thousands of CCGs that regulate many physiological functions including glucose and lipid metabolism, xenobiotic detoxification, hormone secretion, and vascular tone. These rhythms are tissue-specific [48–51], enabling organ-level physiological adaptation. Notably, circadian rhythms undergo age-dependent, tissue-specific remodeling, with aging modifying the identity and the number of rhythmic genes across tissues to varying extents, with declines ranging from 34% in the heart to 75% in the kidney [52,53]. Circadian timing shapes gene expression at multiple levels, including transcriptional, post-transcriptional, and post-translational levels, with the latter being key in driving circadian output in health and chronodisruption [54]. Hence, disruptions—whether genetic, environmental, or behavioral—have substantial molecular and, in fine, adverse cardiometabolic consequences.

**Figure 3:**
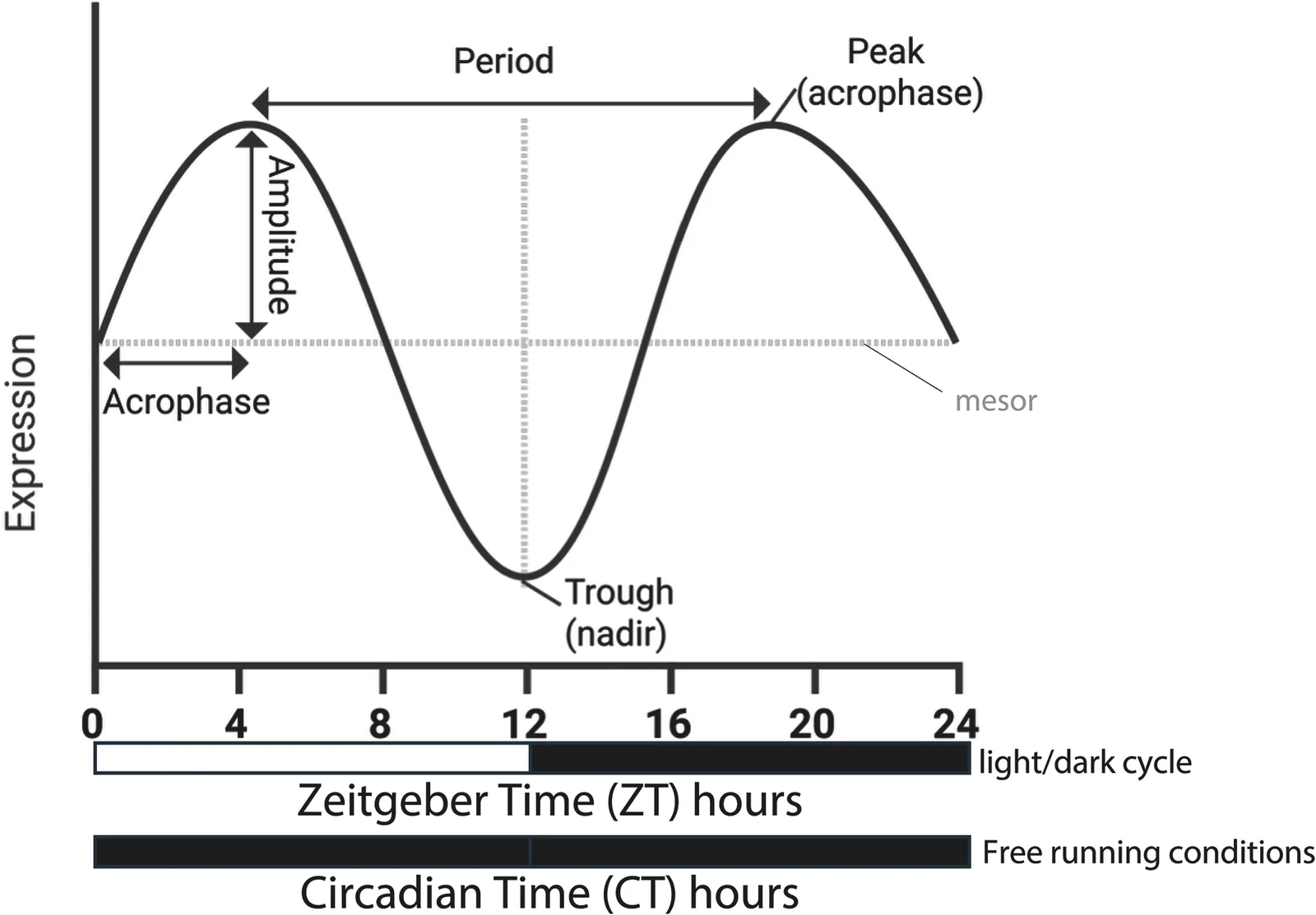
Key parameters of circadian gene-expression rhythmicity A schematic 24-h oscillation illustrating fundamental features of rhythmic gene expression, including acrophase (timing of peak expression), nadir (lowest expression point), amplitude (magnitude of oscillation around the mean), period (time between successive peaks), and mesor (midline estimating statistic of rhythm). ZT time is shown on the *x*-axis, with ZT0 corresponding to lights-on and ZT12 to lights-off under light–dark conditions. Circadian time (CT) represents an internal, endogenous time scale measured under constant conditions (e.g. constant darkness), where CT0 denotes the onset of the subjective day and CT12 the onset of the subjective night. Together, these parameters define the temporal structure of circadian transcriptional rhythms.

### Fine-tuning the clock: auxiliary loops and post-translational modifiers

Beyond the core circadian clock genes, the circadian system relies on additional regulators and post-translational modifiers that enhance its stability and precision (Table 1).

### PARbZIP transcription factors and regulation of clock output genes

D-box regulation further gates CCGs timing through PARbZIP activators (DBP, TEF, and HLF) and the repressor E4BP4 [55], coordinating processes such as hepatic detoxification and immune function. PARbZIP factors and E4BP4 act as opposing D-box regulators of CCGs—PARbZIP enhancing D-box–driven transcription while E4BP4 is repressing it—with partial overlap [56]. Consequently, PARbZIP gene deletion lowers D-box target gene expression while globally preserving rhythmicity in part through compensatory E4BP4 activity, indicating that PARbZIP factors set the amplitude and expression level of D-box regulated genes but have limited influence on rhythmicity [56–58].

### Post-translational ‘timekeeping’

CKIδ/ε phosphorylates PER and CRY [59,60], targeting them for ubiquitination by the F-box proteins FBXL3 [61] and FBXL21 [62] within the SCF E3 ligase complex, leading to proteasomal degradation and determining circadian period length. Reduced CKI activity accelerates PER accumulation and shortens the period, whereas FBXL3 loss-of-function lengthens it by stabilizing CRY. FBXL21, predominant in peripheral tissues, also stabilizes CRY and typically lengthens the period, often counterbalancing FBXL3 [62]. In tissues such as the liver, the NAD^+^-dependent deacetylase SIRT1 modulates CLOCK–BMAL1 by deacetylating PER2 and BMAL1, promoting PER–CRY repression and ending the transcriptional activation phase [63,64].

### Metabolic coupling

AMPK serves as a key energy-sensing regulator of the circadian clock. Low-energy conditions activate AMPK, which phosphorylates CRY1 at sites that lengthen the circadian period [65]. Additional kinases, including CK2 and MAPKs, regulate clock protein stability and nuclear entry, while ubiquitin ligases and SUMOylation further tune clock protein turnover and transcriptional activity (reviewed in [66]). O-GlcNAc signaling transduces nutritional cues into the circadian clock by rhythmically O-GlcNAcylating BMAL1 and CLOCK, a modification that stabilizes the complex by inhibiting its ubiquitination while engaging in phosphorylation crosstalk that fine-tunes CLOCK's transcriptional activity [67,68]. Together, the aforementioned pathways constitute good examples of the high level of integration between metabolic signaling and circadian timing, converting the clock into a multi-layered node integrating environmental and metabolic change.

## Disrupted chronobiology and cardiometabolic risk

Misalignment between the SCN and peripheral clocks destabilizes phase and reduces amplitude of rhythmic physiology, promoting systemic inflammation and neuro-hormonal imbalance. Together, these changes increase the risk of metabolic syndrome, hypertension, dyslipidemia, and cardiometabolic disease. Genetic models define circadian gene function in metabolism, immunity, and cardiovascular physiology (Table 2 and Supplementary Table S1). The following sections examine how disrupted clocks in heart, vasculature, and metabolic organs drive dysregulation of energy balance, vascular integrity, and metabolic homeostasis.

**Table 2:** Representative effects of core circadian clock gene disruption in rodent models of cardiometabolic disease

Gene	Variation	Phenotype	Refs.	Pathology
***Bmal1/Mop3/Arntl***	KO	↓ Blood pressure; ↑ endothelial dysfunction/vascular injury; ↑ thrombosis (aged mice); ↑ hyperlipidemia and atherosclerosis	[69–73]	**Cardiovascular diseases**
	Hepatocyte-KO	In *ApoE^−/−^* mice, ↑ hyperlipidemia; ↑ atherosclerosis.	[72]	
	Myeloid-KO	In *ApoE^−/−^* mice, ↑ atherosclerotic lesion size and ‘M1’ pro-inflammatory macrophages; ↓ ‘M2’ anti-inflammatory macrophages; In *Ldlr^−/−^* mice, ↓ atherosclerotic lesion size and ↓ inflammatory monocyte infiltration; ↑ anti-inflammatory responses.	[73,74]	
	KO	↑ Body fat and obesity; ↑ insulin sensitivity; ↓ hepatic steatosis, fibrosis, and inflammation;	[75]	**Diabetes**
	Pancreatic-KO	↑ Blood glucose (fasting and GTT); ↓ glucose-stimulated insulin secretion; ↓ islet size/proliferation (worsens with age); (↑ ROS/oxidative stress; ↓ mitochondrial function (↑ Ucp2, uncoupling).	[22,76,77]	
	Skeletal-KO	↓ Glucose metabolic gene rhythmicity; ↑ fasted blood glucose; impaired glucose/insulin responsiveness; non-responsive to insulin/AMPK; ↓ fat mass.	[78]	
	Adipocyte-KO	↑ Body weight/fat mass, adipocyte hypertrophy, plasma leptin; ↑ food intake in light phase; ↓ polyunsaturated fatty acids.	[79]	**Obesity**
	Myeloid-KO	↑ Body weight/adiposity with HFD; ↑ macrophage infiltration in white and brown adipose tissue; ↑ systemic inflammation; ↓ energy expenditure/O_2_ consumption; ↓ glucose tolerance; ↓ insulin-induced AKT phosphorylation in adipose, liver, and muscle	[80]	
	T_reg_-KO	↓ Treg fitness/survival in VAT; ↑ visceral adipose tissue inflammation; ↑ lipolysis suppression (loss of diurnal rhythm)	[81]	
	KO	loses circadian white blood cell rhythm	[82]	**Immunity and inflammation**
	Myeloid-KO	↓ Neutrophil recruitment, pulmonary inflammation, alveolar-capillary permeability, injury severity, and mortality (ventilator-induced lung injury model).	[83]	
	Neutrophil-KO	↑ Pulmonary vascular permeability, lung edema, and inflammation; ↓ survival (acute lung inflammation model)	[84]	
	Myeloid-KO	↓ Mitochondrial/oxidative function; ↑ ROS, NF-κB, and pro-inflammatory cytokines (IL-1β, TNF, IL-6); ↓ IL-10; ↑ phagocytosis and motility (RhoA/cytoskeletal remodeling); ↓ weight loss and bacterial burden	[85,86]	
	Hepatocyte-KO	↑ Mortality after LPS	[87]	
***Clock***	*Clock^Δ19^ variant (mutation)*	Disruption of circadian gene expression (multi-tissue); ↓ *Dec1/Dec2*—repressors of the molecular clock (kidney, heart); rhythm preserved in skeletal muscle; ↑ *Npas2* (liver, kidney—compensatory); disrupted diurnal lipid rhythm; ↑ triglycerides; ↑ liver MTP expression → ↑ VLDL secretion.	[88,89]	**Cardiovascular diseases**
		↓ Gluconeogenesis; HFD-amplified diurnal variation in glucose tolerance/insulin sensitivity reversed; ↓ insulin secretion, β-cell proliferation/islet size, rhythmic insulin secretion; ↑ fasting/fed hyperglycemia; ↓ glucose tolerance.	[22,76]	**Diabetes**
		↑ Hyperphagia, body weight/adiposity, hyperlipidemia (triglycerides, cholesterol), hyperleptinemia, hepatic steatosis; ↓ diurnal feeding rhythms (shifted to light phase), energy expenditure; ↑ circadian period length; loss of free-running rhythmicity in constant darkness.	[90,91]	**Obesity**
		↓ Inflammatory cytokines/TNFα-response and ↓ *Per2/Dbp*; ↑ RelB and p100/p52 (non-canonical NF-kB) and ↓ inflammation; ↑ bacterial clearance and sepsis survival, ↓ Acute lung injury (cecal ligation and puncture model); ↑ Cyclophosphamide (CY) sensitivity—↑ weight loss, ↓ lymphocytes, ↑ B-cell depletion.	[92,93]	**Immunity and inflammation**
***Cry1/Cry2***	KO	↑ Salt-sensitive hypertension; ↑ aldosterone (dysregulated adrenal steroidogenesis, ↑ *Hsd3b6*); ↓ plasma renin activity; blunted blood pressure rhythm; hypertension reversed by aldosterone blocker (eplerenone); blunted heart rate/autonomic nervous system rhythms; impaired flow recovery post-hind limb ischemia model; endothelial dysfunction (↓ VEGF signaling, ↓ NO bioavailability)	[94,95]	**Cardiovascular diseases**
	KO	↑ Glucose intolerance (GTT) and fasting hyperglycemia, worsening with age; loss of circadian glucose regulation; disrupted HPA axis; ↓ Glucocorticoid-mediated gene repression, failure to suppress corticosterone during the day	[96]	**Diabetes**
	KO	*Cry1*KO, not *Cry2*KO: ↓ weight gain and fat mass on HFD; *Cry* double-KO: ↑ body weight; ↑ hyperinsulinemia on HFD; abolished circadian rhythm in O_2_ consumption, HR, and body temp → hypermetabolism; ↓ glucose tolerance.	[97–99]	**Obesity**
	KO	↑ IL-6, TNF-α, iNOS (hypothalamus, fibroblasts, macrophages); constitutive ↑ NF-κB, ↑ cAMP-PKA signaling; Bone marrow chimera hematopoietic-intrinsic effect (↑ TNF-α/IL-6 post-LPS); ↑ resistance to cyclophosphamide (CY) (contrasts with CY-hypersensitive *Clock^Δ19^/Bmal1*KO).	[93,100]	**Immunity and inflammation**
***Per1/Per2***	*Dahl Salt-Sensitive Per1* KO	↑ Blood pressure and heart rate with high-salt diet; loss of circadian blood pressure synchrony; ↓ renal function (↓ creatinine clearance).	[101]	**Cardiovascular diseases**
	*Per2* mutation	↑ Aortic endothelial dysfunction; ↓ NO production; ↓ vasodilatory prostaglandins; ↑ cyclooxygenase-1-derived vasoconstrictors; ↓ angiogenesis and vascular repair; ↑ endothelial cell senescence; ↓ blood-flow recovery post-ischemia; ↑ autoamputation; WT EPC infusion/BM transplant rescues blood-flow recovery and prevents autoamputation.	[102,103]	
	Myeloid-specific *Per1/2* double KO	↑ Insulin resistance; ↑ adipose and liver inflammation; ↑ NF-κB, IL-1β, IL-6, TNF-α in myeloid cells	[104]	**Diabetes**
	*Per2* mutation	Retina: ↓ eNOS/NO signaling; ↑ acellular capillaries, permeability, tight-junction disruption; ↑ pro-fibrotic (TGF-β1). Bone marrow: ↓ BMPC mobilization and proliferation; ↓ nerve fiber density (neuropathy)—phenotype occurs without hyperglycemia	[105]	
	*Per1* KO	Severely disrupted locomotor rhythms in constant darkness; SCN clock gene rhythms unaffected (unlike *Per2* KO); ↓ peak PER2/CRY1 protein	[106]	**Obesity**
	*Per2* KO	HFD: loss of diurnal glucocorticoid rhythm, disrupted feeding rhythm (equal light/dark intake); ↓ α-MSH rhythm, restored by α-MSH injection (→ weight loss); ↑ LDL-C, liver index, *Pparα/Cyp7a1/Cpt1*.	[107,108]	
	Myeloid cell- *Per1/Per2* Double KO	Exacerbates diet-induced adipose and liver inflammation and systemic insulin resistance; ↑ M1 (inflammatory) macrophages; ↑ IL-1β/IL-6/TNF-α and ↓ PPARγ (macrophages)	[104]	**Immunity and inflammation**
	*Per2* KO	Resistance to LPS-induced endotoxic shock; ↓ IFNγ and IL-1β (NK/NKT cells); no change in TNF-α, IL-6, or IL-10; ↓ intestinal amino acids; disrupted hepatic clock genes; ↑ hepatic inflammation (*Il-1β, Il-6, Tnf-α, Myd88, Nf-κB p65*); ↑ antioxidant capacity (↑ SOD, GSH-Px; ↓ MDA)	[108,109]	
	*Per2^Brdm1^* mutation	↓ TLR9-induced TNF-α/IL-12 in macrophages; disrupted TLR9 circadian expression in splenic macrophages and B cells; CLP sepsis severity is circadian/TLR9-dependent; vaccination is more effective at peak TLR9 responsiveness	[110]	

**Abbreviations used:** KO: knockout, KD: knockdown, Tg: transgenic, HFD: high-fat diet, NO: nitric oxide, GTT: glucose tolerance test.

Cardiovascular diseases include atherosclerosis, dyslipidemia, hypertension, thrombosis, aortic aneurysm; Diabetes includes hyperglycemia and hypoinsulinemia.

Obesity includes adiposity and insulin resistance.

### Obesity, adiposity, and insulin resistance

Obesity, a polygenic, multifactorial disease, is characterized by excessive adiposity and is a major cardiometabolic risk factor. WAT is a dynamic endocrine organ of adipocytes and stromal vascular cells, including progenitor cells, immune cells, and endothelial cells, that stores energy and modulates systemic metabolism via adipokines and cytokines (reviewed in [111]). Beige and brown adipose tissue (BAT) depots harbor intrinsic functions governing adipogenesis (the formation of mature adipocytes), lipogenesis (lipid storage), lipolysis (lipid breakdown), thermogenesis (heat production), and endocrine signaling.

SNP variants in core clock genes link rhythmic disruption to obesity and insulin resistance. In 537 Europeans, 3 common *CLOCK* haplotypes (∼30% frequency)—CAT, TGT, and CGC (rs4864548, rs3736544, and rs1801260)—associate with adiposity traits [112]. rs4864548 correlates with greater waist circumference and obesity, while rs1801260 associates with lower BMI, waist-hip ratio, and leptin. *CLOCK* rs4864548A and rs1554483G increased overweight/obesity risk by 1.8-fold [113]. Among 1495 overweight/obese Europeans, carriers of the rs1801260 minor C allele had shorter sleep, higher ghrelin, delayed mealtimes, poorer Mediterranean diet adherence, and greater resistance to weight loss [114]. In the Genetics of Lipid Lowering Drugs and Diet Network (GOLDN) cohort, rs3749474 minor alleles associate with 1.33-fold higher energy intake, suggesting impaired *CLOCK* environmental responsiveness [115]. *PER2* variants (rs2304672C>G, rs4663302C>T) correlate with abdominal obesity and poor Mediterranean diet adherence in 454 overweight/obese subjects (83.7% female) [116]. In contrast, *REV-ERBα* rs2314339 was associated with abdominal obesity via reduced physical activity rather than energy intake [117]. Notably, these human genetic associations are consistent with findings from circadian mutant mouse models. For example, *CLOCK* variants associated with adiposity, altered eating behavior, and obesity [113,114] parallel the obesity, hyperphagia, disrupted feeding rhythms, and metabolic syndrome observed in *ClockΔ19* mice [90,118]. Similarly, *PER2* variants linked to abdominal obesity and metabolic syndrome traits [116] are concordant with studies showing that *Per2* deficiency disrupts lipid metabolism, feeding rhythms, and susceptibility to diet-induced obesity in mice [107,119,120]. Mechanistically, most of these variants are non-coding (intronic or promoter-proximal) polymorphisms rather than protein-altering mutations and are therefore likely to act by modulating clock-gene transcript abundance, splicing, or transcription-factor binding (e.g. E-box or CRE elements) rather than by disrupting protein structure, consistent with their generally modest, population-level effect sizes relative to the more severe phenotypes seen with complete gene loss (reviewed in [121]). Accordingly, recent analyses show that genetic variants can influence the rhythmicity of hundreds of genes in a tissue-specific manner, potentially influencing this way disease pathogenicity and response to treatments [122,123].

As discussed, eating at atypical times disrupts the circadian system, increases weight gain, and increases obesity risk [124]. Consequently, aligning feeding schedules with circadian rhythms can restore clock function and improve metabolic outcomes (reviewed in [124]). In a 20-week trial, late eaters lost less weight than early eaters despite similar calorie intake [125]. Shift workers, who often eat irregularly, show higher obesity prevalence (reviewed in [126]). Sleep restriction alters leptin and ghrelin, promoting hunger and metabolic imbalance that raises obesity and diabetes risk [127]. High-fat diet (HFD)-induced or genetic (*ob/ob*) obesity in rodents lowers *Bmal1* and *Rev-erbα* in the central nervous system (CNS) and up-regulates PPARα, a key regulator of lipid metabolism, in the obese brainstem, while increasing *Clock* and decreasing *Cry1* in peripheral tissues [128].

WAT stores triglycerides, while BAT dissipates energy via thermogenesis. Mouse adipose tissue shows robust circadian regulation: ∼4% of WAT and ∼8% of BAT transcripts oscillate, including core clock genes (*Bmal1, Clock, Per1/2/3, Cry1/2*, and *REV-ERBα/β*) and clock-controlled genes (*Dbp, E4bp4, Stra13*, and *Id2*) [129]. Human subcutaneous WAT shows ∼2% gene rhythmicity [130]. Active phase time-restricted feeding restores *Bmal1* oscillation, enhances creatine-mediated futile cycling, and increases thermogenesis, mitigating obesity without caloric restriction [131]. Interestingly, misaligned feeding shifts WAT circadian clock in accordance with feeding time, whereas the BAT molecular clock remains entrained to the light–dark cycle yet exhibits internal desynchronization of metabolic oscillations, highlighting a fundamental divergence in how these adipose depots integrate nutrient timing and circadian control [39]. Further, insulin acutely entrains the adipose circadian clock by rapidly inducing clock genes including *PER2* and triggering phase shifts in circadian oscillations, thereby establishing insulin as a direct molecular link between feeding and adipose clock resetting [132].

Manipulation of circadian gene expression reveals their metabolic roles (Table 2). *Bmal1* increases during adipocyte differentiation and lipogenesis while inhibiting the WNT signaling pathway, which otherwise suppresses adipogenesis [133]. Kettner et al. [134] show that BMAL1/CLOCK establishes circadian leptin transcription in adipose tissue and that circadian disruption—whether genetic or environmental—induces leptin resistance by uncoupling central and peripheral clock control of energy homeostasis, effects that are markedly sex-dependent. These findings align closely with adipocyte-specific *Bmal1* deletion models, in which *Bmal1* loss leads to obesity, adipocyte hypertrophy, hyperleptinemia, disrupted feeding rhythms, reduced polyunsaturated fatty acids, impaired thermogenesis, and lower energy expenditure under HFD conditions [131,134]. This disruption of the adipose clock shifts feeding toward the usual rest phase and alters polyunsaturated fatty acid composition, which modifies hypothalamic neuropeptide expression and feeding behavior [79]. Conversely, adipocyte-overexpression of *Bmal1* enhances rhythmicity, thermogenesis, energy expenditure, and glucose tolerance, protecting against diet-induced obesity [131].

Among circadian-deficient animal models, the *ClockΔ19* mutation [91] shows a defect in circadian behavior that later work linked to profound metabolic dysregulation, demonstrating that disruption of CLOCK-driven rhythmicity leads to obesity through impaired timing of feeding and energy balance. *ClockΔ19* mutants show hyperphagia, loss of feeding rhythms, increased adiposity, adipocyte hypertrophy, triglyceride accumulation, reduced lipolysis, hyperlipidemia, hyperleptinemia, and hepatic steatosis, with strain-dependent severity [90,118]. *Cry1/2* double-knockout mice gain fat and body mass despite reduced food intake on an HFD, showing enlarged white adipocytes, up-regulation of lipogenic genes, and smaller lipid droplets in interscapular brown fat, suggesting impaired brown adipogenesis [97]. *Per* genes exert nuanced roles. *Per1* knockout mainly affects locomotor activity and age-related decline in constant darkness without major SCN transcript changes [106,135]. *Per2*-deficiency perturbs lipid metabolism and drives diet-induced obesity, abolishes diurnal glucocorticoid rhythmicity, and disrupts feeding rhythms in part by eliminating rhythmicity of α-MSH (alpha-melanocyte-stimulating hormone), a key appetite regulator [107,119]. α-MSH restores feeding rhythms and prevents obesity [107]. PER2 also represses PPARγ-dependent adipogenesis, and loss of *Per2* alters lipid metabolism and circulating lipids [120,136]. Glucocorticoids repress *Per3* to promote adipogenesis, whereas *Per3* overexpression inhibits adipocyte differentiation [137]. Consequently, *Per3* knockout increases adiposity and reduces muscle mass [137].

REV-ERBα integrates circadian timing and thermogenesis. *Rev-erbα* knockout increases *Ucp1* expression in BAT and cold tolerance but disrupts temperature rhythms and increases macrophage infiltration via CCL2 (MCP-1) [7,138]. Overexpression in skeletal muscle or pharmacological activation (e.g. agonists SR9009 or SR9011) boosts mitochondrial biogenesis, fat oxidation, and energy expenditure, improving exercise endurance and reducing adiposity [139]. However, SR9009 and SR9011 are not solely REV-ERBα specific and may exert off-target effects, including mitochondrial dysfunction in certain cell types, which should be considered when interpreting pharmacological results [140]. REV-ERBα activation promotes adipogenesis, while deficiency increases fat accumulation and metabolic dysfunction under HFD [141]. *Rorα* deficiency elevates UCP1 levels and thermogenesis in WAT and BAT, promotes browning of white adipose via PGC1α and PRDM16, and confers resistance to diet-induced obesity [142].

Immune clocks also matter. Myeloid-specific *Bmal1* deletion worsens HFD-induced obesity, increases macrophage infiltration in WAT and BAT, and drives systemic inflammation (IL-1β, IL-6, IFNγ, and CCL2) with impaired energy expenditure and insulin signaling across adipose, liver, and muscle tissues [80]. Treg lymphocyte-specific *Bmal1* deletion disrupts diurnal lipolysis and promotes visceral adipose inflammation [81]. Collectively, these studies demonstrate cell-type-specific clock control of adipose biology, energy balance, thermogenesis, and immunometabolism.

### Type 2 diabetes mellitus

Type 2 diabetes (T2D) is characterized by chronic hyperglycemia arising from impaired pancreatic β-cell insulin secretion associated with insulin resistance in muscle, liver, and/or adipose tissue. Under normal conditions, insulin activates PI3K-AKT signaling and GLUT4 translocation to promote glucose uptake in muscle and adipose tissue (reviewed in [143]). In T2D, muscle glucose uptake declines, hepatic gluconeogenesis increases, and adipose tissue releases excess fatty acids that worsen insulin resistance. The condition is strongly associated with obesity, particularly visceral fat accumulation, which drives chronic low-grade inflammation and hormonal imbalances that impair insulin signaling. Glucose tolerance and insulin sensitivity follow circadian rhythms—post-prandial peaks after midday with reduced β-cell responsiveness and insulin sensitivity later in the day [144–146]—and glucagon and glucagon-like peptide-1 display similar rhythmicity [147,148], underscoring a strong link between circadian timing and glycemic control.

Genetic variation in circadian clock genes contributes to T2D susceptibility. Given that excess adiposity (BMI >27) strongly increases diabetes risk [149], obesity-linked variants in *CLOCK*, *PER2*, and *REV-ERBα* likely also modulate T2D susceptibility. Further, a *CLOCK* haplotype (rs3749474/rs4580704/rs1801260) associates with lower insulin resistance due to preserved rhythmic expression of glucose-uptake pathway genes involved in glucose uptake [112]. In a Greek cohort, *BMAL1* variants, rs7950226 and rs11022775, are linked to gestational diabetes susceptibility, although the underlying mechanistic link to β-cell or placental clock function remains to be established [150]. *CRY1* rs2287161 reduces insulin sensitivity by altering CRY1 stability; the minor C allele increases fasting insulin and HOMA-IR insulin resistance in Mediterranean (*n* = 728) and North American (*n* = 820) cohorts [151]. In a Finnish cohort of ∼1000 individuals, *PER2* rs934945 and *NPAS2* rs2305160 were associated with increased waist circumference, triglycerides, and fasting glucose, and in obese visceral adipose tissue the same variants showed altered expression correlating with metabolic-syndrome traits—together indicating adipose-clock disruption that disturbs adipokine rhythms and promotes insulin resistance [152,153]. Clinically, *CRY* and *PER* variants are associated with impaired glucose tolerance and stress-related overeating through weakened repression of CLOCK–BMAL1. Across multiple large GWAS, *CRY2* (rs11605924 A-allele) and the melatonin receptor gene *MTNR1B* (rs10830963 G-allele) repeatedly emerge as top glycemic-trait loci, with the *CRY2* risk allele increasing 2-h glucose and the *MTNR1B* G-allele raising fasting and post-challenge glucose, thereby elevating T2D risk [154–157]. These findings—together with trans-ancestry analyses identifying *CRY2* and *MTNR1B* among the most robust genomic contributors to glycemic dysregulation—underscore that circadian-gene variants exert directionally adverse effects on glucose homeostasis.

Short-term sleep loss (<6 h/night) or reduced REM sleep impairs insulin sensitivity and elevates diabetes risk by disrupting circadian and melatonin signaling [158]. GWAS similarly link *CRY2* rs11605924 and melatonin receptor gene (*MTNR1B*) variants (rs1387153, rs10830963) to higher fasting glucose and HOMA-IR, the latter with a strong effect on fasting glucose (β = 0.1 mmol/l per G allele, *P* = 4.2E−35), consistent with evidence that enhanced MTNR1B-mediated melatonin signaling suppresses nocturnal insulin secretion and reduces β-cell responsiveness, thereby increasing future T2D risk [154,159]. Human islets from individuals carrying the *MTNR1B* risk allele showed higher *MTNR1B* expression and reduced insulin release, confirming that heightened melatonin signaling directly impairs β-cell function and promotes hyperglycemia, in line with melatonin’s physiological role in inhibiting nocturnal insulin secretion [156]. Collectively, circadian clock gene variants—modulated by adiposity, diet, sleep, and melatonin signaling—converge on β-cell function, hepatic gluconeogenesis, and adipose endocrine timing to shape glycemic control and T2D risk.

In mice, global *Bmal1* knockout causes hyperglycemia and reduced circulating insulin, while glucose tolerance and whole-body insulin sensitivity were improved [75,76,160]. Disruption of *Clock* or *Bmal1* impairs pancreatic β-cell transcriptional programs, reducing insulin synthesis and secretion and leading to hypoinsulinemia, glucose intolerance, and diabetes [22]. Pancreatic *Bmal1* loss induces hypoinsulinemia, defective glucose-stimulated insulin secretion (GSIS), oxidative stress, mitochondrial dysfunction, and progressive β-cell failure, with growing evidence that β-cell resilience to these insults itself differs by sex [77,161]. Muscle-specific deletion reduces GLUT4, glycolytic flux, and insulin responsiveness [78], while CLOCK knockdown in human primary skeletal myotubes reduces glucose uptake [162]. Insulin activates hepatic AKT to phosphorylate BMAL1 at Ser42, causing BMAL1 to dissociate from DNA, bind 14-3-3, and exit the nucleus, thereby resetting peripheral clock rhythms—revealing a molecular mechanism by which postprandial insulin signaling (which is dysregulated in T2D) directly alters circadian timing and may contribute to diabetic clock–metabolic misalignment [163]. The *ClockΔ19* mutation causes obesity, metabolic syndrome, hyperglycemia with defective β-cell exocytosis, impaired GSIS, smaller islets, hepatic lipid accumulation, and disrupted feeding rhythms [22,90].

Disruption of *Cry* or *Per* genes dismantles the clock’s negative limb and the repression of the CLOCK–BMAL1 complex, desynchronizing tissue-specific programs that govern insulin action, substrate storage, and inflammation. *Cry1/2* double knockouts develop fasting hyperglycemia, severe glucose intolerance, insulin resistance, and loss of 24-h metabolic rhythmicity [96]. HFD accelerates the above obesity and metabolic disruption [97]. CRY1 normally suppresses hepatic gluconeogenesis by limiting cAMP and *Pck1/G6pc* expression, and its degradation under HFD promotes morning hyperglycemia [164]. *Cry1* deletion prevents diet-induced obesity, whereas *Cry2* loss produces milder effects, highlighting CRY1’s protective metabolic role [98]. Consistently, stabilizing CRY1 pharmacologically via KL001 suppresses gluconeogenesis and glucagon-induced glucose production [165]. *Per1/2* knockout increases visceral adiposity, adipose NF-κB-dependent inflammation, and systemic insulin resistance, while myeloid *Per1/2* loss further amplifies macrophage-driven inflammation in adipose tissue and liver, exacerbating whole-body metabolic dysfunction [104]. Mechanistically, *Per2* mutants have reduced insulin clearance and blunted insulin-receptor and PI3K–AKT signaling in peripheral tissues (liver, adipose, heart) and resultant impaired peripheral glucose disposal [166]. They also show reduced glycogen synthase, lower fasting glycogen, and disrupted hepatic nutrient partitioning.

In pancreatic β-cells, REV-ERBα is rhythmically expressed and required for glucose-stimulated insulin secretion and β-cell proliferation, while its silencing or diabetogenic stress impairs insulin release via REV-ERBα-mediated autophagy inhibition [167]. In α-cells, REV-ERBα regulates glucagon secretion, with reduced expression blunting low-glucose-induced release and agonism enhancing calcium signaling and glucagon output [168]. In the liver, REV-ERBα represses gluconeogenesis and hepatic glucose production, highlighting its role as a circadian brake on glucose output [169]. Within the SCN, REV-ERBα/β in GABAergic neurons set the daily rhythm of insulin-mediated suppression of hepatic glucose production; SCN-specific deletion causes waking-time glucose intolerance and reduced insulin sensitivity, which are rescued by restoring REV-ERB rhythmicity, paralleling the human ‘extended dawn’ phenomenon [170,171].

### Cardiovascular diseases

The cardiovascular system is tightly governed by circadian regulation, with blood pressure, heart rate, vascular tone, and myocardial metabolism all displaying robust 24-h rhythms. These follow a characteristic daily pattern—rising in the morning and gradually declining through the evening, reaching their lowest levels during sleep [172]. Disruption of these rhythms, whether behavioral, environmental, or genetic, consistently increases cardiovascular morbidity in both humans and rodent models, underscoring the circadian clock as a central regulator of cardiovascular health. A striking illustration of gene-environment interplay is the *tau* mutant Syrian hamster, whose shortened intrinsic period (22 h) desynchronizes it from the standard 24-h light–dark cycle: *tau/+* heterozygotes housed under normal 24-h lighting develop severe cardiomyopathy, fibrosis, contractile failure, and renal disease with early death, whereas housing under a 22-h T-cycle matched to their genotype restores rhythmic behavior and reverses the cardiorenal phenotype, demonstrating that misalignment itself, rather than clock-gene dysfunction *per se*, can be sufficient to drive organ pathology [173].

Genetic variation in core circadian genes contributes to cardiovascular risk, though the field remains less explored. *BMAL1* variants show functional relevance: *BMAL1* rs3816358 predicts non-dipping blood-pressure patterns in young-onset hypertension across a 372-person discovery and 619-person replication cohorts [174], while *BMAL1* rs11022775 protects against dyslipidemia in African Americans [175]. *CLOCK* polymorphisms similarly influence cardiometabolic outcomes. *CLOCK* rs4580704 lowers T2D incidence and reduces stroke risk in T2D patients within the 7098-participant PREDIMED trial [176], whereas *CLOCK* rs6850524 increases hypertension risk in Hispanic/Latino populations [175]. Additional *CLOCK* variants rs13113518, rs35115774, and rs6832769, and *CRY1* rs2078074 influence intestinal cholesterol absorption in 456 Europeans, linking circadian variation to lipid-related cardiovascular pathways [177]. In contrast, *CRY2* rs2292912 increases dyslipidemia risk in African Americans [175], while rs11605924 shows a nominal sleep-duration interaction for HDL-C regulation, with each additional hour of sleep associated with a 0.01 mmol/l HDL-C increase [178]. *PER2* rs11894491 associates with LDL-C levels in Europeans [177], and rs6431590 predicts nocturnal non-dipping blood-pressure patterns, a phenotype linked to elevated cardiovascular complications [177]. Finally, sex-specific effects are evident for *REV-ERBα* rs2071570, where boys, but not girls, showed significantly lower total cholesterol, LDL-C, and ApoB levels, with AA male carriers showing the most favorable lipid profiles in a cohort of 1268 children [179].

In mice, global *Bmal1* knockout leads to lower arterial pressure and heart rate and impairs endothelial function and sympathoadrenal tone (reduced catecholamines) characterized by abolished circadian hematopoietic stem cell egress by flattening stromal CXCL12 rhythms. These changes heighten vascular injury, blunt remodeling, increase collagen deposition and age-related thrombosis, and finally accelerate arterial injury and atherogenesis [69–71]. Circadian disruption also drives a prominent dyslipidemia via altered hepatic lipid flux—increased VLDL/ApoB secretion (MTP–ApoB axis) and reduced biliary cholesterol disposal (clock control of bile-acid/cholesterol pathways such as CYP7A1)—thereby driving plaque growth [72]. Hepatocyte-specific *Bmal1* deletion recapitulates, whereas hepatocyte overexpression reverses these lipid/atherosclerotic phenotypes. Myeloid *Bmal1* functions are context dependent: in *ApoE^−/−^* mice it enlarges lesions via increased Ly6C^hi^ monocyte recruitment, M1 polarization, and pro-inflammatory cytokines, consistent with clock loss unleashing glycolytic, NF-κB–biased programs [73], while in *Ldlr^−/−^* mice, myeloid *Bmal1* deletion reduces plaque and aortic aneurysm burden by damping leukocyte infiltration without altering systemic metabolism or blood pressure, underscoring background- and lipid-milieu specificity [74].

BMAL1 also governs blood-pressure rhythms: whole-body *Bmal1* knockout rats show reduced mean arterial blood pressure but retain diurnal blood-pressure rhythm [180], whereas smooth-muscle-specific *Bmal1* deletion in mice blunts circadian blood-pressure dipping [181]. Tissue- and sex-specific effects further characterize blood pressure regulation by the clock. Renal collecting-duct *Bmal1* deletion lowers blood pressure in male mice [182,183]. The adrenal gland clock also directly contributes to these phenotypes: adrenal-specific *Bmal1* deletion alters blood-pressure rhythm and the timing of feeding behavior [184], while loss of adrenal *Bmal1* augments stress- and ACTH-induced glucocorticoid responses in a sex-specific manner, being most pronounced in females [185]. This is consistent with the broader pattern of male-biased circadian cardiovascular vulnerability. Perivascular adipose *Bmal1* regulates resting-phase blood pressure via angiotensinogen [186], while feeding/light cues set diurnal blood-pressure rhythms through both *Bmal1*-dependent and -independent pathways [187]. Chronic circadian stress also impairs blood pressure and sodium homeostasis in a diet- and sex-specific manner [188]. Similarly, *Per1* loss disrupts renal sodium-transport genes and blood-pressure control [189,190] and drives sex-dependent arterial stiffening and hypertrophic remodeling [191]. *Per2* deficiency sensitizes mice to angiotensin-II-dependent hypertension under low-salt conditions [192]. Together, these findings point to dissociable, sex-specific renal, vascular, and adipose clock mechanisms underlying blood-pressure regulation. Beyond blood pressure, BMAL1 also directly shapes cardiac structure and metabolism. In the heart, cardiomyocyte-specific *Bmal1* dampening does not materially change feeding and activity behavior but shifts fuel use toward fatty-acid oxidation with decreased glucose utilization—an inappropriately ‘fasted-like’ program persisting even when fed [193]. By contrast, global *Bmal1* knockout animals develop early dilated cardiomyopathy with left ventricular mass increase via an increase in cell number, later wall thinning, and contractile failure, linked to BNIP3 reduction resulting in excess mitochondrial degradation [194], phenotypes mirrored in *BMAL1*-deficient human cardiomyocytes [195]. Interestingly, females are generally less affected and partly protected from age-dependent cardiomyopathy [196,197]. Overall, *Bmal1* loss broadly promotes vascular inflammation, dyslipidemia-driven atherosclerosis, and cardiac dysfunction, yet phenotypes diverge by cell type, genetic background, sex, and hemodynamic context.

The *ClockΔ19* variant disrupts circadian gene expression across metabolic and cardiovascular tissues, producing a coordinated loss of physiological rhythmicity [198]. This disruption features reduced *Dec1/Dec2* expression in kidney and heart, preserved skeletal-muscle oscillations, and compensatory *Npas2* up-regulation in kidney and liver [88]. These molecular shifts impair diurnal lipid control, producing sustained hypertriglyceridemia through increased hepatic MTP expression and excess VLDL secretion [89]. Cardiovascular phenotypes further illustrate the systemic impact of circadian failure: *ClockΔ19* mice exhibit cardiomyocyte hypertrophy, increased heart mass, diminished contractility, and loss of rhythmic PTEN, while other core clock-gene knockouts present with salt-sensitive hypertension, dysregulated aldosterone, blunted diurnal blood-pressure rhythms, and endothelial dysfunction with impaired VEGF and nitric-oxide signaling [196]. Although female *ClockΔ19* mice show partial resilience, maintaining myocardial glucose uptake and resisting age-related dysfunction, both sexes demonstrate elevated AKT activation, indicating a shared pro-hypertrophic drive [197]. Male *ClockΔ19* mice develop the most pronounced pathology, consistent with disrupted PTEN–AKT rhythmicity. Interpretation remains limited by the lack of tissue-specific *ClockΔ19* models, which constrains separation of direct cardiac effects from systemic circadian disruption. Later studies using cardiomyocyte-specific *Clock* mutants or cardiomyocyte *Bmal1* knockouts show that diurnal cardiac metabolism and energy handling rely directly on the intrinsic peripheral cardiac clock [193,199,200].

*Cry1/Cry2* double-knockout mice develop pronounced salt-sensitive hypertension due to adrenal steroidogenic dysregulation—elevated aldosterone, reduced plasma renin, blunted blood-pressure, and impaired VEGF-nitric oxide endothelial signaling that limits ischemic blood-flow recovery; hypertension is fully reversed by an aldosterone inhibitor [94,95]. Similarly, *Per1*-null Dahl salt-sensitive rats show exaggerated high-salt hypertension, renal injury, and loss of circadian regulation of sodium-handling and blood-pressure genes [101]. *Per2* mutants develop severe endothelial dysfunction with reduced nitric oxide and prostaglandins, increased COX-1 vasoconstrictors, and impaired angiogenesis [102]. Further, *Per2* mutation accelerates vascular aging and severely impairs endothelial-progenitor-cell function, leading to reduced nitric-oxide signaling, defective angiogenesis, and poor recovery from ischemic injury [103]. Consistent with these vascular phenotypes, *Per2* mutation in hypercholesterolemic mice markedly enlarges atherosclerotic plaque burden and elevates IL-6, demonstrating that PER2 loss synergistically worsens cardiovascular-disease progression by amplifying inflammation and circadian dysfunction [201].

*Rev-erbα* knockout suppresses CYP7A1, reducing bile-acid synthesis, elevating SHP and E4BP4, and causing dyslipidemia with phase-shifted rhythms [202,203]. In cardiomyocyte-specific *Bmal1* knockouts, *Rev-erbα/β* mesor and rhythmicity expression collapse, and the *Rev-erb* agonist SR9009 partially rescues cardiac hypertrophy, fibrosis, and rate-pressure defects, although *Rev-erbα/β* double-knockout studies reveal additional REV-ERB-independent actions [204]. Consistent with a cardioprotective role for REV-ERB, a single post-ischemia-reperfusion dose of the REV-ERB agonist SR9009 suppresses cardiac NLRP3 inflammasome activation, predominantly in cardiac fibroblasts, reducing immune cell infiltration, limiting infarct expansion, and preventing progression to heart failure in an REV-ERB-dependent manner, with maximal treatment efficacy observed when it coincides with the phase of REV-ERB cardiac expression [205]. Loss of cardiomyocyte *Rev-erbα/β* also elevates E4BP4, suppressing NAD^+^ salvage and disrupting diurnal cardiac metabolism, effects blunted by nicotinamide riboside in females [206,207]. RORα provides further cardioprotection by limiting ischemia–reperfusion injury and angiotensin-II-induced hypertrophy and cell death [208,209]. Together, these findings establish that circadian-clock disruption, whether through genetic variation, gene deletion, or altered downstream regulators, acts as a unifying driver of cardiovascular pathology by destabilizing metabolic, vascular, endocrine, and myocardial homeostasis.

### Immunity and inflammation

The circadian clock regulates immune function, producing daily rhythms in immune-cell trafficking, cytokine release, and host defense. Immune cells contain intrinsic clocks synchronized by the central pacemaker via hormonal and neural signals, creating time-of-day differences in infection risk, vaccine responses, and inflammatory and cardiometabolic disease activity [210,211]. Because inflammation drives obesity, T2D, and cardiovascular disease, circadian clock-gene variants implicated in these disorders may also affect inflammatory pathways. For example, *CLOCK* rs4580704 is linked to increased insulin sensitivity, lower T2D risk, and reduced inflammation [176]. Variants in *PER2* have been associated with autoimmunity: rs2304674 with rheumatoid arthritis and rs11894491 with systemic lupus erythematosus (reviewed in [121]). However, due to variability and complexity in inflammatory marker phenotyping and pathology, relatively few studies have explicitly examined associations between clock-gene SNPs and inflammation.

BMAL1 is essential for circadian control of immune and inflammatory responses across multiple cell types. Global *Bmal1* deletion abolishes daily oscillations of circulating white blood cells and bone-marrow CXCL12 production, with cortisol only partially restoring hematopoietic rhythms [82]. Cell-specific loss of *Bmal1* produces divergent immune phenotypes: myeloid-specific *Bmal1* knockout reduces neutrophil recruitment, pulmonary inflammation, and mortality in ventilator-induced lung injury [83], whereas neutrophil-specific deletion increases the formation of neutrophil extracellular traps, pulmonary permeability, lung edema, and mortality during acute lung inflammation [84]. Macrophage *Bmal1* deficiency drives profound metabolic and inflammatory reprogramming, impaired mitochondrial oxidative metabolism, increased ROS and NF-κB signaling, elevated inflammatory cytokines IL-1β, TNF, and IL-6, and heightened mortality from LPS-induced sepsis [85,86,212,213]. Hepatocyte-specific *Bmal1* deletion similarly increases LPS-induced mortality by disrupting FXR signaling and the feeding-cycle-dependent metabolic protection against sepsis [87]. Together, these findings highlight BMAL1 as a central regulator of rhythmic immunity, inflammatory tone, and host resilience to infection and inflammatory injury.

The *ClockΔ19* mutant shows impaired NF-κB-related immune responses and disrupted circadian gene expression [214]. *ClockΔ19* mutant MEFs display reduced pro-inflammatory cytokine induction (IL-6, CXCL1, IL-1β) after LPS administration and diminished TNF responsiveness and lower *Per2* and *Dbp* expression, indicating blunted inflammatory activation. Similarly, *Cry1/2* double-knockout mice show heightened basal inflammation, with increased IL-6, TNF, iNOS, elevated NF-κB activity, and augmented cAMP–PKA signaling across multiple tissues [100]. Bone-marrow chimera studies confirm a hematopoietic origin of this pro-inflammatory phenotype. *Per1* knockout reduces and dampens age-dependent glucocorticoid rhythms and alters circadian profiles of NK-cell effector molecules such as IFN-γ and granzyme B, alongside disrupted *Per2* and *Bmal1* rhythms in NK cells [215]. Myeloid-specific *Per1/2* double knockout exacerbates diet-induced adipose and hepatic inflammation and systemic insulin resistance, driven by increased pro-inflammatory M1 macrophage accumulation and elevated inflammatory cytokines [104]. Consistently, *Per2* deficiency confers resistance to LPS-induced endotoxic shock due to reduced IFN-γ and IL-1β production by NK and NKT cells, despite largely preserved TNF, IL-6, and IL-10 responses [109]. However, *Per2* loss also disrupts hepatic clock gene expression, impairs amino-acid metabolism, and promotes hepatic inflammation through elevated *Myd88* and NF-κB signaling while enhancing antioxidant capacity [108]. The *Per2^Brdm1^* mutation disrupts TLR9-mediated cytokine responses and increases sepsis severity in a circadian-dependent manner, reflecting the underlying daily rhythms of TLR9 expression in macrophages and B cells [110]. Consistent with this, vaccination timed to the circadian peak of TLR9 responsiveness produces markedly stronger adaptive immune responses, and in a TLR9-dependent sepsis model, disease severity varies according to the timing of septic challenge, aligning with the oscillation of TLR9 expression [110].

*Rev-erbα* knockout mice lose circadian gating of cytokine and chemokine production, showing time-of-day–independent increases in LPS-induced IL-6, IL-12p40, CCL5, CXCL1, and CCL2 [213]. Across organs, *REV-ERBα* deficiency enhances NLRP3 inflammasome activation, elevates IL-1β and IL-18, and promotes excessive neutrophil and macrophage recruitment. These changes manifest as severe colitis [216], increased lung neutrophilic inflammation with loss of rhythmic CXCL1/2 and G-CSF [217], and heightened susceptibility to LPS/D-GalN–induced fulminant hepatitis with greater hepatic infiltration, injury, and mortality [218]. Hepatic ischemia-reperfusion injury is similarly worsened, with elevated ALT/AST, inflammatory cytokines, myeloperoxidase activity, and myeloid cell accumulation [219]. In the CNS, *Rev-erbα* loss induces hippocampal microgliosis, astrocyte activation, NF-κB signaling, oxidative-stress pathways, and heightened neuroinflammation. In *Rev-erbα* knockout mice, the diurnal rhythm of microglial activity is abolished, leading to increased complement-mediated phagocytic activity, excessive synaptic pruning, and loss of hippocampal, particularly CA3, synaptic connectivity [220]. Cell-specific deletion confirms its role in timing inflammatory responses (myeloid cells) and restraining pulmonary inflammation (bronchial epithelium), with more severe effects when REV-ERBα and REV-ERBβ are both absent [217].

Pharmacological activation of REV-ERBα consistently suppresses inflammatory signaling across multiple tissues. In human macrophages, the REV-ERB agonist GSK4112 suppresses LPS-induced IL-6 secretion without inhibiting IL-8 and increasing intracellular heme, with hemin similarly suppressing LPS-induced IL-6 induction [213,217]. The REV-ERB inverse agonist GSK1362 disrupts REV-ERBα interactions with repressive co-regulators (NCoR1, SMRT2, and RIP140), relieves REV-ERB-mediated repression of *BMAL1* transcription, stabilizes REV-ERBα protein against inflammation and cytokine-driven degradation, and thereby contributes to restraining pulmonary inflammatory responses [217]. In the liver, REV-ERBα activation with SR9009 down-regulates NLRP3 inflammasome activity and reduces caspase-1 activation and IL-1β and IL-18 production, leading to diminished macrophage infiltration, attenuated hepatocellular injury, and improved survival in LPS/D-GalN-induced fulminant hepatitis in a *Rev-erbα*-dependent manner [218]. Similar hepatoprotective effects are observed in ischemia–reperfusion models, where the REV-ERB agonist SR9009 attenuates liver damage, lowering plasma ALT and AST levels, histological damage, and myeloperoxidase activity—and these improvements coincide with suppressed NLRP3 inflammasome activation, reduced IL-1β, IL-6, and TNF production, and decreased neutrophil and macrophage accumulation [219]. In the CNS, REV-ERBα agonists suppress LPS-induced hippocampal neuroinflammation, lowering IL-6 and CCL2 release and inhibiting NF-κB activation [220]. Collectively, these findings show that pharmacological activation of REV-ERBα exerts broad anti-inflammatory, anti-inflammasome, and tissue-protective effects across peripheral and central immune compartments.

In contrast with REV-ERBα, RORα promotes inflammatory cytokine expression and increases susceptibility to immune responses. Classic work in staggerer (*Rora^sg/sg^*) mutant mice, which carry a spontaneous deletion removing part of the RORα ligand-binding domain, producing a truncated, non-functional protein rather than a discrete point mutation [221], shows that mutation of *Rorα* leads to aberrant immune responses and exaggerated inflammatory injury, including increased vulnerability to lung inflammation, establishing RORα as a driver of heightened innate immune tone [222]. More recent mechanistic studies show that RORα supports the expression of NF-κB–dependent cytokines in macrophages and T cells, with *Rorα*-sufficient cells exhibiting stronger pro-inflammatory activation, whereas *Rorα* deficiency dampens LPS-induced cytokine release and reduces endotoxic shock responses [223].

*Nfil3* (*E4bp4*) knockout mice exhibit widespread defects across innate and mucosal immunity (extensively reviewed in [224]), but their association with the specific role of E4BP4 as a circadian output transcription factor is still unclear. They show severely impaired NK cell development, with loss of mature NK cells and markedly reduced NK-mediated cytotoxicity [225]. In the intestine, *Nfil3* deficiency leads to exaggerated inflammation and impaired mucosal immunity, driven by increased macrophage IL-12/23 production and heightened inflammatory responses to gut bacteria. Similarly, IBD patients show reduced *NFIL3* expression, and colitis-prone mice fail to induce *Nfil3* in response to microbiota [226]. NFIL3 is also essential for CD8^+^ conventional dendritic cell (cDC1) development: knockout mice display reduced cDC numbers, defective CD8^+^ T-cell cross-priming, diminished IL-12 production upon TLR3 stimulation, and reduced *Batf3* expression, a key transcription factor for cDC1 lineage commitment [227]. Beyond these defects, *Nfil3*-deficient mice show a broad loss of innate lymphoid cells (ILC1, ILC2, and ILC3) across tissues, increasing susceptibility to gut infections such as *C. rodentium* and *C. difficile*, along with reduced NK and LTi-like cells necessary for proper lymphoid tissue formation [226]. Myeloid cells in knockout mice are hyper-inflammatory, with elevated IL-12p40 production and increased macrophage activation, contributing further to chronic mucosal inflammation [228]. In summary, the circadian clock orchestrates rhythmic immune and inflammatory responses across tissues, with core clock genes—such as *BMAL1, CLOCK, PER1/2, REV-ERBα, RORα, and NFIL3*—shaping cytokine production, immune-cell development, host defense, and susceptibility to inflammatory and cardiometabolic disease.

## Sex differences in chronobiology

Circadian sex differences reflect intrinsic clock properties and sex-steroid effects on the SCN and its photic/non-photic inputs (extensively reviewed [229,230]). As noted above, most knockout phenotypes described in preceding sections were established in males only. This section synthesises the smaller body of work examining sex as a biological variable in clock function. Estrogen enhances photic sensitivity and accelerates re-entrainment, whereas androgens dampen clock-gene transcription. Mechanistically, estrogen directly regulates *Per2* expression and rhythmicity: estrogen induces *Per2* transcription and modulates the period of its circadian oscillation in reproductive and other estrogen-responsive tissues [231,232], while PER2 in turn promotes ERα degradation, forming a reciprocal feedback loop between the clock and estrogen signaling [231]. This estrogen–PER2 crosstalk offers a plausible molecular basis for the higher-amplitude, female-biased clock-gene rhythms described below. The female SCN uniquely gates the oestradiol-dependent preovulatory luteinizing hormone surge [233], which is absent in males and after ovariectomy. Women typically have slightly shorter intrinsic circadian periods (≈24.09 h versus ≈24.19 h), resulting in earlier phases of core body temperature minimum, cortisol rhythms, and melatonin onset, along with higher melatonin amplitude (∼38%–41%) and greater nocturnal-light-induced melatonin suppression [234].

Sex differences in clock function arise from at least two dissociable sources: gonadal hormone exposure and sex chromosome complement itself. Using the four-core-genotypes mouse model, which uncouples chromosomal sex from gonadal phenotype, gonadal females show larger photic phase delays than gonadal males regardless of chromosomal complement, while androgen-receptor expression in the SCN and behavioral photic responsiveness are independently shaped by XX/XY genotype, indicating that sex chromosome genes contribute to circadian phenotypes beyond hormonal programming alone [235].

At the molecular level, female mice exhibit higher amplitude transcriptional rhythms in the liver, with nearly twice as many rhythmic genes as males—an enrichment consistent with estrogen-linked regulation and accompanied by sex-biased patterns in core clock gene expression and phase (e.g. higher amplitudes of *Bmal1* or *Cry1*) [236,237]. Similar differences were reported in humans [50]. Female rhythmic liver genes are enriched for lipid-metabolic, apoptotic, and ribosomal-biogenesis pathways, whereas male rhythmic genes are enriched for protein transport, biosynthesis, and transcription programs, and these sex-specific rhythmic patterns are partly microbiome-dependent [236–239]. Germ-free mice lose hepatic sexual dimorphisms via effects on sexual development, growth hormone signaling and xenobiotic receptor activation [237,240]. In shift-work models, females retain metabolic rhythmicity and preserve glucose homeostasis, whereas males lose rhythmicity and develop glucose intolerance [239,241,242]. These male-specific metabolic deficits can be partly rescued by antibiotic depletion of the microbiota or by castration [243]. Human data likewise show that women maintain higher amplitude rest-activity rhythms and more consolidated sleep, whereas men are more susceptible to circadian misalignment, including higher rates of metabolic syndrome and diabetes under shift work [234,244]. Misalignment also produces sex-divergent hormonal responses—raising leptin in men but increasing ghrelin in women—leading to opposite shifts in appetite despite similar energy expenditure [244].

Women show stronger parasympathetic ‘rest-and-digest’ activity, whereas men exhibit greater overall heart rate variability [245]. In mice, adrenal *Bmal1* deletion disrupts circadian control of aldosterone and sodium handling in both sexes, but females retain more rhythmic sodium-excretion patterns, whereas males show greater disruption [246]. In rats, whole-body *Bmal1* deletion lowers mean arterial pressure but leaves circadian blood pressure rhythms intact in both sexes, while only males lose the normal diurnal rhythm of sodium excretion, indicating that females preserve renal circadian function and have greater physiological resilience [180]. Female mouse hearts also show more robust rhythmic gene expression, and cardiomyocyte-specific *Bmal1* deletion markedly diminishes sex differences in cardiac transcriptional programs, including changes in key transcription factors such as GATA4 and TBX5, underscoring the key role of sex-specific heart gene regulation [247]. *Clock* mutant male mice develop severe age-related cardiomyopathy, while females are protected unless ovariectomized, demonstrating estrogen-dependent buffering of circadian-related cardiac aging [197].

As discussed in the ‘Cardiovascular disease’ section above, this male-biased pattern recurs across independent genetic models spanning the renal-adrenal-cardiac axis: whole-body and kidney-specific *Bmal1* deletion preferentially affects male blood-pressure rhythms and sodium handling. Kidney-specific *Bmal1* deletion lowers baseline blood pressure in male but not female mice [182,183], and cardiomyocyte-specific *Bmal1* deletion markedly reduces sex-specific cardiac gene expression [247], indicating a broader role for BMAL1 in establishing sex differences in the heart beyond blood pressure. Similarly, *Per1* loss produces male-specific non-dipping hypertension in response to high-salt/mineralocorticoid challenge, with males developing a blunted nocturnal blood-pressure dip and renal sodium-handling defects, whereas female *Per1*-null mice are protected from this non-dipping phenotype [190,248]. The convergence of these findings across distinct genes, tissues, and physiological endpoints, rather than any single tissue-specific mechanism, points to estrogen-dependent buffering of clock-gene function as a unifying explanation for male-biased cardiovascular vulnerability. This interpretation parallels human epidemiology: the menopausal decline in estrogen removes a key driver of clock-gene rhythmicity and coincides with rising cardiovascular risk [249] and a high prevalence of non-dipping nocturnal blood pressure in perimenopausal and postmenopausal women [250]. Further, irregular male shift workers exhibit sex-specific cardiac dysfunction, including reduced left-ventricular function, higher heart rate, and prolonged electrical recovery time between beats (corrected QT interval), highlighting male vulnerability to circadian disruption in humans [251].

Sex differences also extend to adiposity and appetite regulation. Global *Bmal1*-null mice show markedly greater fold-increases in body adiposity in females than males (2.3-fold versus 1.8-fold on a normal diet), despite comparable arrhythmic clock-gene expression in adipose tissue and liver in both sexes, indicating that downstream metabolic sensitivity to clock disruption, not just the molecular lesion itself, is sex-dependent [252]. This is echoed in humans: circadian misalignment lowers the satiety hormone leptin and raises the hunger hormone ghrelin in women, while producing the opposite hormonal shift in men, despite similar total energy expenditure between sexes [244]. Moreover, sex differences also shape β-cell resilience and diabetes risk. Compared with male islets, female pancreatic islets, in both mice and humans, better maintain glucose-stimulated insulin secretion under endoplasmic-reticulum stress and in the context of T2D, consistent with the lower T2D prevalence in pre-menopausal women [253]. While this mechanism has not yet been directly linked to clock-gene function, it aligns with, and may partly explain, the male-biased glucose intolerance seen under circadian misalignment described above.

Females also mount stronger time-of-day-dependent immune responses, including larger IL-6 surges or greater CD4+ T cell activation, reflecting sex differences in autonomic rather than purely hormonal regulation, and may underlie women's increased susceptibility to certain autoimmunity [254,255]. Interestingly, parental exposure to dim light at night before conception produces lasting sex-specific immune alterations in offspring: maternal exposure impairs fever responses and increases inflammatory IL-1 production in male offspring, while paternal exposure reduces TNF expression and bactericidal capacity in offspring of both sexes [256]. These findings show that circadian disruption prior to conception can reprogram offspring immunity in parent- and sex-specific ways, even when offspring develop under normal light–dark cycles.

Taken together, these findings show that sex is not a peripheral variable but a systematic modifier of circadian clock function across every organ system discussed in the present review, from adipose and β-cell resilience to cardiac, renal, and adrenal physiology. Mechanistically, this dimorphism appears to arise from at least two converging layers: sex-steroid–clock crosstalk at the molecular level (e.g., estrogen–PER2 feedback) and tissue-autonomous differences in stress resilience (e.g., islet ER-stress handling) that are independent of, but compounded by, circadian misalignment.

## Therapeutic opportunities

Most clinical trials targeting circadian pathways have focused on jet lag, sleep disorders, or behavior interventions such as melatonin use, time-restricted feeding, and scheduled exercise (reviewed in [257]). In NCT02204735, obese individuals consuming more calories earlier in the day lost more weight [258], and in NCT03553524, afternoon interval training improved 24-h glucose levels in T2D [259], highlighting the value of aligning behavior with biological time.

Time-restricted eating (TRE) is an emerging dietary strategy that improves metabolic health by aligning food intake with circadian rhythms rather than focusing solely on calorie restriction. TRE limits eating to a 4- to 10-h daily window, inducing a fasting period of 14 to 20 h. Across 2 to 12 months, TRE typically produces 3%–5% weight loss (mainly fat mass), driven mainly by a reduction in food intake [260,261]. Early TRE, which aligns eating with earlier circadian phases, appears particularly effective. In a 14-week trial, early TRE combined with calorie restriction produced greater reductions in body fat, improved insulin sensitivity, and enhanced glycemic control compared with calorie restriction alone [262]. A six-month study in adults with T2D also found that an 8-h TRE schedule (12–8 PM) resulted in 3.6 kg weight loss and a 0.9% reduction in HbA1c versus controls [263]. Evidence indicates that TRE's efficacy increases when fasting exceeds 14 h [264]. Importantly, responses vary by chronotype; individuals with later sleep–wake preferences may benefit most when meal, sleep, and activity timing are gradually shifted earlier [265]. Collectively, these findings highlight behavioral chronotherapy as a promising, nonpharmacological approach to enhance metabolic health, though larger long-term trials are needed to refine optimal timing and durability of effects.

Two therapeutic strategies are now emerging: (i) small molecules that modulate core circadian clock components and (ii) chronotherapy that times treatment to better suit endogenous rhythms, including potential sex-specific timing differences. Small-molecule approaches aim to adjust period, phase, or amplitude to improve glucose regulation, thermogenesis, lipid metabolism, blood pressure, and immune tone. CRY stabilizers such as KL001 (CRY1) and CRY2-selective analogues (KL101, TH301, and SHP656) lengthen circadian period, suppress glucagon-stimulated gluconeogenesis, improve glucose clearance in obese mice, and promote BAT thermogenesis with increased *Ucp1* expression [165,266,267]. Casein kinase 1 inhibitors (Longdaysin, PF-670462, and PF-4800567) lengthen the circadian period by enhancing PER2 nuclear retention [268]. Because PER regulates daily rhythms in glucose handling, vascular tone, and blood pressure, stabilizing PER2 may help correct early-phase advances seen in sleep and cardiometabolic disorders. In contrast, GSK3β inhibitors reduce PER2/CRY2 phosphorylation, shortening period and promoting phase advances that may blunt early-morning surges in glucose and blood pressure [269]. However, GSK3β is a highly pleiotropic kinase with central roles in Wnt and insulin signaling, neuronal survival, and other pathways, raising substantial concerns around off-target effects and toxicity that limit its attractiveness as a selective chronotherapeutic target [270]. Targeting nuclear receptors also yields metabolic and anti-inflammatory benefits. REV-ERB agonists (GSK4112, SR9009, and SR9011) repress BMAL1-driven transcription, improving glucose and lipid homeostasis, increasing energy expenditure, promoting fat mass loss in diet-induced obesity, and reducing inflammation [139,213,271]. ROR agonists such as nobiletin enhance Clock amplitude, increase physical activity, and induce weight loss in *db/db* mice [272]. Collectively, these chemically diverse agents modulate circadian timing and amplitude to influence pathways central to cardiometabolic health.

Chronotherapy aligns treatment with biological rhythms to enhance efficacy and/or reduce toxicity. In chronic kidney disease, nocturnal hypertension and a nondipper blood-pressure profile are common. These are better corrected by bedtime dosing of anti-hypertensives such as angiotensin-converting enzyme inhibitors or angiotensin receptor blockers, which more effectively lower sleep-time blood pressure, restore dipping, and reduce cardiovascular risk [273]. The HYGIA Chronotherapy Trial reported large cardiovascular benefits with bedtime antihypertensive dosing [274], but these findings remain controversial given the null results of the more rigorous TIME trial [275]. This divergence likely reflects methodological differences rather than a true absence of chronotherapeutic effect: HYGIA was a single-center, open-label PROBE-design study with cardiovascular hazard ratios that many statisticians consider implausibly large for a dosing-time intervention, raising concerns about its methodological rigor, whereas TIME was a large, multicenter, blinded-endpoint trial with self-reported dosing time and a considerably lower event rate, which may have limited its power to detect a modest true effect. Notably, chronotype-matched sub-analyses of TIME and the more recent BedMed trial suggest dosing time may still matter for some individuals: aligning antihypertensive timing with personal chronotype, such as morning dosing for ‘larks,’ evening/bedtime dosing for ‘owls,’ was associated with fewer cardiovascular events than mismatched timing in both cohorts [276,277], suggesting chronotype-informed dosing may better capture circadian therapeutic benefit than fixed clock time. Meta-analyses suggest bedtime aspirin may reduce platelet activity and lower blood pressure, but evidence is weak and requires confirmation in larger trials [278]. Because cholesterol synthesis peaks at night in humans, short-half-life statins (e.g. simvastatin) lower LDL cholesterol more effectively when taken in the evening [279], whereas evidence for long-half-life statins (e.g. atorvastatin) is inconsistent [280,281]. Together, these findings indicate that circadian-based therapies offer promising mechanistic and clinical opportunities but require more rigorous evidence before widespread cardiometabolic implementation.

## Future directions and clinical implications

Advancing circadian biology in cardiometabolic disease requires resolving key mechanistic and clinical uncertainties. Establishing causality—beyond human association studies linking circadian misalignment with obesity, diabetes, and dyslipidemia—remains essential. Future work must clarify how peripheral clocks across metabolic tissues coordinate and how behaviors such as unhealthy diet, poor diet schedule, sleep loss, and inactivity interact with intrinsic rhythms to drive disease. Clinically, the promise of chronotherapy is tempered by mixed evidence. Optimizing the timing of antihypertensives, glucose-lowering drugs, and statins will require well-powered, long-duration trials that align treatment with individual physiological rhythms rather than fixed clock time. Next, personalized chronomedicine represents a major opportunity. Integration of continuous data from wearable devices with predictive algorithms could identify optimal dosing windows and feeding schedules, while parallel efforts to discover circadian biomarkers will be critical for tailoring interventions. Importantly, AI-enabled temporal phenotyping now provides proof-of-concept that circadian disease subtypes—such as atrial fibrillation chronophenotypes identified through unsupervised analysis of 24-h Holter ECG profiles—can be discovered directly from real-world physiological data, offering a transformative pathway for clinical translation. These findings show that chronophenotypes can inform time-based monitoring and therapeutic strategies, enabling optimized ECG-monitoring schedules, more accurate risk assessment, and future chronotherapy approaches aligning medication administration or ablation timing with each patient's specific rhythmic vulnerability [282]. Finally, translation into practice must address broader societal disruptors such as shift work, artificial light exposure at night, and 24-h food access, while accounting for variability across age, sex, genetics, and chronotype. Aligning therapies and environments with the body's intrinsic clock offers a promising path to improving cardiometabolic health.

## Supplementary Material

Supplementary Table S1

## Open Access

Open access for this article was enabled by the participation of Monash University in an all-inclusive Read & Publish agreement with Portland Press and the Biochemical Society under an agreement with CAUL.

## Abbreviations

AMPK: AMP-activated protein kinase
AVP: arginine vasopressin
BMAL1: brain and muscle aryl hydrocarbon receptor nuclear translocator-like protein 1
BAT: brown adipose tissue
CKIδ/ε: casein kinase Iδ/ε
CT: circadian time
CLOCK: circadian locomotor output cycles kaput
CCGs: clock-controlled genes
CRY: cryptochrome 1–2
E4BP4: E4 binding protein 4
GSIS: glucose-stimulated insulin secretion
GSK3β: glycogen synthase kinase 3 beta
HFD: high-fat diet
NPAS2: neuronal Per-Arnt-Sim (PAS) domain protein 2
PARbZIP: proline and acidic amino acid-rich basic leucine zipper proteins
PAS: Per-Arnt-Sim
PER: period 1–3
REV-ERB: reverse-erythroblastosis virus α/β
ROR: retinoic acid receptor-related orphan receptor
SIRT1: sirtuin 1
SCN: suprachiasmatic nuclei
TRE: time-restricted eating
TTFLs: transcriptional–translational feedback loops
T2D: type 2 diabetes
VIP: vasoactive intestinal polypeptide
WAT: white adipose tissue
ZT: Zeitgeber
